# Supplementary material for: Seeding hESCs to achieve optimal colony clonality
Source: Sci Rep. 2019 Oct 25;9:15299. doi: 10.1038/s41598-019-51897-0 (PMC6814789; doi:10.1038/s41598-019-51897-0)
Supplement: Supplementary file 1 — Seeding hESCs to achieve optimal colony clonality: Supplementary Information [file 41598_2019_51897_MOESM1_ESM.pdf]

Seeding hESCs to achieve optimal colony clonality

L E Wadkin, S Orozco-Fuentes, I Neganova, S Bojic, A Laude, M Lako, N G Parker, A Shukurov

Supplementary Information

Model parameters

For the simple deterministic model, equation (1), given in the main text, rewrite $N(t) = N_0 e^{\gamma t}$ where $\gamma \sim \text{Norm}(\mu, \sigma^2)$ as $N(t) = e^{\log(N_0)+\gamma t}$.
Since γ follows a Normal distribution, $X \equiv \gamma t + \log(N_0) \sim \text{Norm}(\text{t}\mu + \log(\text{N}_0), \text{t}^2\sigma^2)$ through linear transformation properties of Normal random variables.Therefore the exponential of the variable follows a Lognormal distribution with the same parameters by definition of a lognormal random variable, $e^X \sim \text{Norm}(\mu_0, \sigma_0{}^2)$ where $\mu_0 = t\mu + \log(N_0)$ and $\sigma_0 = t\sigma$.

Single population model

Assuming that all colonies begin as single cells, i.e. $N_0 = 1$, maximum likelihood parameter estimation gives the fitting to the single population exponential growth model $N(72) \sim \text{LogNorm}(3.08, 0.56^2)$. The model based on the experimental data therefore becomes equation (1) with $\mu = 0.0428\,\text{h}^{-1}$ and $\sigma = 0.0077\,\text{h}^{-1}$, corresponding to a doubling time of approximately 16 hours. The experimental data with the model fitting is shown in Figure S1. The overall shape of the distribution is roughly captured but the bimodal nature is not. It is worth noting that when simulated stochastically this single population model can result in a bimodal distribution for $N(t)$ given the probabilistic nature of the stochastic simulations, see Figure S1, but this is a rare occurrence happening around once in 100 simulations for 50 colonies. Note that simulating from equation (1) will always recover exactly the theoretical distribution when ran for large enough numbers of colonies.

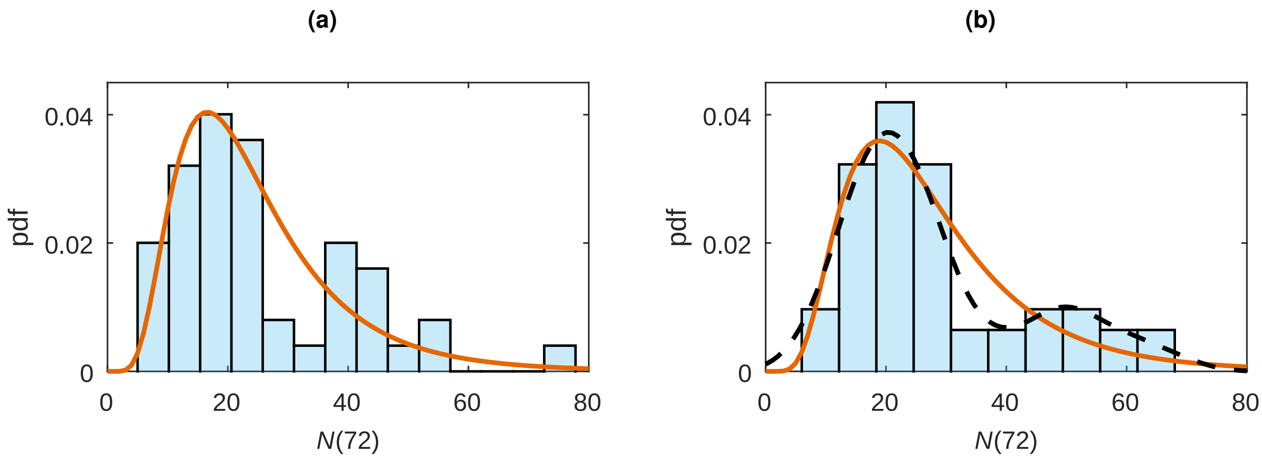

Figure S1. (a) Experimental $N(72)$ histogram with the fitting obtained from model 1, $N(72) \sim \text{LogNorm}(3.08, 0.56^2)$ fitting. (b) An example simulation from model (1) for 50 colonies at 72 hours producing a bimodal distribution, with kernel density estimation in black dashed, and the theoretical LogNormal(3.08, 0.56^2) fitting in orange.

Two population model with identical growth rates

In the main text we present a mixture fitting to the experimental data allowing us to extract the growth parameters for two populations. We assume the growth rates for each population are different, meaning the two groups differ in both their initial condition and growth rate. Here we present the results if we assume that the two growth rates for each population are the same,

$$\begin{cases} N_\text{A}(t) = e^{\gamma t},\ \gamma \sim \text{Norm}(\mu, \sigma^2), & \text{with probability } \alpha, \\ N_\text{B}(t) = 2e^{\gamma t},\ \gamma \sim \text{Norm}(\mu, \sigma^2), & \text{with probability } \beta. \end{cases} \quad (1)$$

The number of cells then follows a lognormal distribution, $N(t) \sim \text{LogNorm}(\mu_0, \sigma_0{}^2)$, where $\mu_0 = t\mu + \log(N_0)$ and $\sigma_0^2 = t^2\sigma^2$ as presented above. Here the two peaks of $N(72)$ will be caused by the differing initial condition only. The fittings obtained are $N_\text{A}(72) \sim \text{LogNorm}(2.79, 0.37^2)$ and $N_\text{B}(72) \sim \text{LogNorm}(3.49, 0.37^2)$, with the probabilities $\alpha = 0.72$ and $\beta = 0.28$. Therefore, we have $\mu = 0.0388\,\text{h}^{-1}$ and $\sigma = 0.0052\,\text{h}^{-1}$. The results of the fitting are shown in Figure S2,

alongside the main text mixture fitting for comparison. This fitting does not predict the pairs of cells population well. From this we can conclude it is appropriate to consider the two populations as having different growth rates.

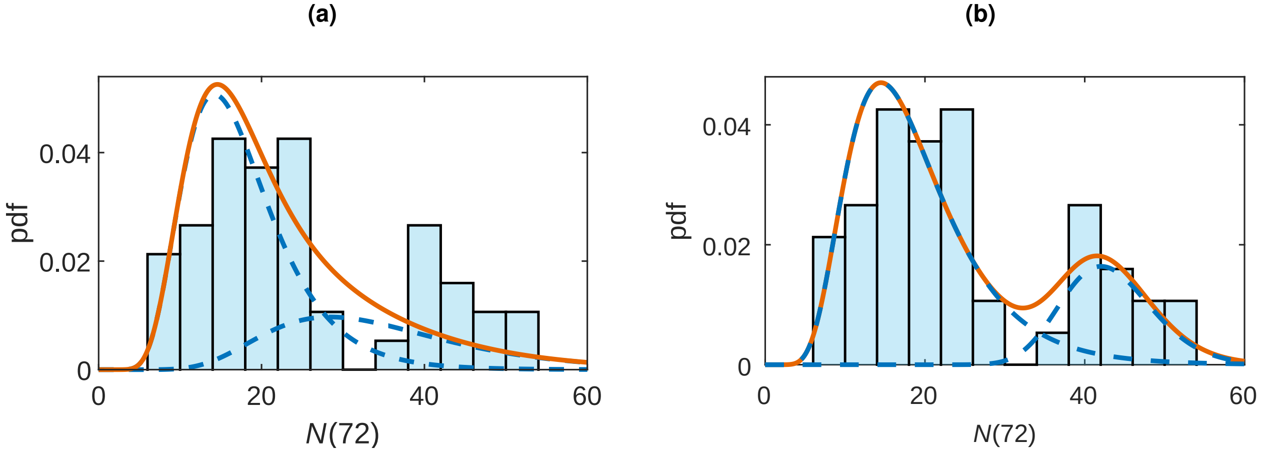

Figure S2. (a) The mixture fitting assuming the growth rates for the two populations are the same. The population fittings, $N_{\mathrm{A}}(72) \sim \mathrm{LogNorm}(2.79, 0.37^2)$ with mixture probability 0.72 and $N_{\mathrm{B}}(72) \sim \mathrm{LogNorm}(3.49, 0.37^2)$ with mixture probability 0.28 are shown in blue dashed, and the overall mixture distribution in orange. (b) The mixture fitting from the two population model assuming the growth rates are different.
